# Automated evaluation of cardiac contractile dynamics and aging prediction using machine learning in a *Drosophila* model

**DOI:** 10.21203/rs.3.rs-2635745/v1

**Published:** 2023-03-21

**Authors:** Aniket Pant, Yash Melkani, Girish C. Melkani

**Affiliations:** 1 Department of Pathology, Division of Molecular and Cellular Pathology, Heersink School of Medicine, University of Alabama at Birmingham, AL 35205, USA; 2 Department of Materials Science and Engineering, Georgia Institute of Technology, GA 30332, USA; 3 Engineering Physics Department, College of Engineering, University of California, Berkeley, CA 94720, USA

**Keywords:** Machine learning, medical segmentation, cardiovascular disease, *Drosophila* heart model, aging prediction

## Abstract

The *Drosophila* model has proven tremendously powerful for understanding pathophysiological bases of several human disorders including aging and cardiovascular disease. Relevant high-speed imaging and high-throughput lab assays generate large volumes of high-resolution videos, necessitating next-generation methods for rapid analysis. We present a platform for deep learning-assisted segmentation applied to optical microscopy of *Drosophila* hearts and the first to quantify cardiac physiological parameters during aging. An experimental test dataset is used to validate a *Drosophila* aging model. We then use two novel methods to predict fly aging: deep-learning video classification and machine-learning classification via cardiac parameters. Both models suggest excellent performance, with an accuracy of 83.3% (AUC 0.90) and 77.1% (AUC 0.85), respectively. Furthermore, we report beat-level dynamics for predicting the prevalence of cardiac arrhythmia. The presented approaches can expedite future cardiac assays for modeling human diseases in Drosophila and can be extended to numerous animal/human cardiac assays under multiple conditions.

## Introduction

Cardiovascular disease (CVD) continues to be among the leading causes of death and disability in the United States and a major public health burden. Despite other risk factors, aging is one of the major risk factors for advancing CVD. As the modern human population is increasingly adapting to lifestyles that mimic shift work further exacerbates CVD risks^1^. The *Drosophila melanogaster* (fruit flies) model system will allow us to comprehensively examine the potential cardiac benefit of a simple lifestyle modification and identify the relevant molecular and physiological changes. Gaining pathophysiological insights is essential for designing therapies and in vivo models including *Drosophila* models and modern technologies including machine learning are powerful tools for understanding several aspects of human pathophysiology, including aging and CVD. Despite some morphological and functional differences, *Drosophila* and human hearts conserved pathways appear to govern form and function^2–9^. Several genetic and non-genetic risks for heart diseases in humans also increase disease risks in *Drosophila* during aging. Moreover, mutant flies carrying genetic variants that are associated with a higher risk for CVD result in a similar outcome in the fly^3,4,6^. Furthermore, flies like humans living in industrial societies, consume some of their daily caloric intake at night and nutritional challenges that compromise cardiac function in humans have similar effects in flies^10–12^.

Progress in the high-speed recording of optical cardiac imagery has led to remarkable development in the application of *Drosophila*, zebrafish, and embryonic mouse heart for cardiac modeling^13^. For example, current canonical methods using Semi-Automatic Optical Heart Analysis (SOHA) software offers methods for measuring relevant cardiac parameters during aging and under multiple disease conditions^13^. However, the SOHA software requires manual selection of points of interest in diastole and systole phases, allowing the software to track changes in heart morphology. Moreover, Dong et. *al* demonstrated the use of 3D convolutional architectures for segmentation of *Drosophila* heart images studied via optical coherence microscopy (OCM) setups^14^. The authors reported robust segmentation (92% IoU) of OCM videos, with capabilities for calculating EDD, ESD, heart area and heart rate. Similarly, Lee *et. al* demonstrate *Drosophila* heart-beat counting using segmentation of optical coherence tomography (OCT) recordings^15^. They demonstrate similar segmentation of OCM videos and also apply PCA procedure for heart morphology reconstruction. Work by Klassen et *al.* enabled *in vivo* imaging of *Drosophila* aging models with a fluorescence imaging and conventional image-processing techniques for high-resolution, unanesthetized beating patterns^16^. Using a conventional computer vision approach, the authors provide methods for automatic segmentation and parameter (chamber diameter, fractional shortening, systolic interval, cardiac output, and heart wall velocity) calculations. We find much discussion of successful automatic analysis techniques for use with optical coherence microscopy but find a lack of literature surrounding similar techniques in standard high-resolution optical microscopy setups, likely due to added complexity in visualized heart morphology.

Through literature review, we find that a limited amount of cardiac physiological data has been analyzed using machine learning from morphological data collected with OCT or OCM techniques, with quantification of all relevant cardiac parameters. Moreover, heart analyses using machine learning techniques in the literature have not tested rigorous *Drosophila* aging or disease models. Furthermore, there is a lack of automated methods for analysis of high-speed *Drosophila* cardiac optical recordings in general optical microscopy setups. Modern video volumes outgrow the use of manual analysis techniques, necessitating automatic analysis methods. Our method differs from others in that it allows for automatic analysis of optical cardiac recordings at high spatial and temporal resolutions. Furthermore, we are the first to provide all cardiac statistics via a deep learning assisted pipeline. We attempt a solution with a well-known deep learning-assisted medical image segmentation architecture, the UNet. Recent developments in machine learning have enabled state-of-the-art dense image segmentation platforms, such as the development of full convolutional networks (FCNs)^17^, SegNets^18^, and UNets^19–22^. Historically, UNet architectures have been applied for segmentation cell nuclei, spleen, and liver segmentation^23,24^, COVID-19 prognosis^25^, and more. Human heart analysis has been a central focus in medical segmentation efforts ^26–28^. In 2020, Ouyang *et. al*^26^ demonstrated the use of convolutional architectures for segmentation and beat-level analysis of human echocardiograms. They demonstrate real-time prediction of ejection fraction with comparable or less variance than that of human predictions. In this work, we draw inspiration from similar applications of medical image segmentation and apply UNet architectures to segmentation and beat-level analysis of *Drosophila* cardiac recordings.

To augment human analysis of *Drosophila* cardiac recordings, we apply techniques in medical segmentation for analysis of high-speed optical recordings. We calculate multiple diagnostic cardiac parameters for assessing cardiac function from videos alone. A 2D attention-based UNet architecture is applied for frame-level dense segmentation of cardiac recordings. Using only the generated annotations, we calculate beat-level cardiac parameters and beating patterns. Cardiac parameters are calculated on a per-beat resolution. We used *n* = 54 hearts for model training and validated an experimental aging model with *n* = 177 hearts. This deep-learning approach to *Drosophila* cardiac analysis facilitates robust analysis of *Drosophila* cardiac physiology and enables access to future downstream analysis techniques. We investigate the use of both Drosophila cardiac statistics and cardiac videos for fly age prediction as an example downstream task. We determine that conventional machine learning and deep learning methods can be applied for predicting fly age, suggesting that morphological features evident in video recording can be an effective predictor of an aging phenotype.

## Results

### Overview of presented machine learning pipeline

**Figure 1:**
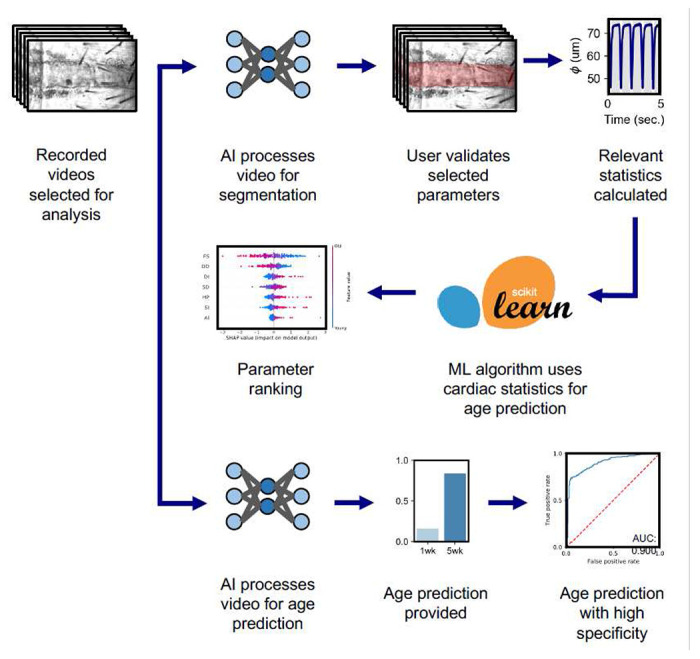
Proposed deep learning pipeline. One pipeline enables frame-level segmentation of heart walls, while two alternate pipelines are used for age classification. For the segmentation task, users upload videos for analysis, perform segmentation using a trained neural network, select validated parameters, and extract beat-level parameters. The middle pipelines enable age-prediction of hearts via calculated cardiac statistics, further enabling transparent modeling of physiological parameters. Lastly, the bottom-most pipeline details an age-prediction task using a neural network, thus providing an alternate method for age-prediction.

Analysis of *Drosophila* high-speed cardiac recordings requires high-throughput and accurate measurement techniques. Current methods involve tedious analyses with large amounts of personnel hours, necessitating automatic next-generation methods. In this work, we propose three machine learning enabled pipelines that is well-suited for rapid analysis of *Drosophila* cardiac recordings. Depicted in Fig. 1, we establish two unique tasks: (1) heart wall detection via semantic segmentation and (2) age prediction. In the presented segmentation pipeline (Fig. 1), a user begins by uploading high-speed cardiac recordings to a central workstation via a file-transfer protocol. After this, the user selects videos for segmentation and analysis and provides them to our segmentation model, which is made accessible via a PyTorch interface^29^. Then, using our segmentation model, heart wall regions are tagged on a frame-by-frame basis, allowing us to easily calculate average heart diameter and area per-frame. After results have been generated per frame, users may vary network parameters such as selecting a region-of-interest in diameter calculations and a model confidence threshold. Supplementary Fig. 1 (.mp4) demonstrates a video-format output of our network results for a given heart, for five seconds. Once a satisfactory segmentation is acquired, users may export time-resolved morphological data. Finally, using the exported morphological data, we make available relevant cardiac statistics for assessing function and rhythmicity. Example Python notebooks are provided for segmenting and analyzing heart videos with easy to use, interactive user-interfaces.

Two separate pipelines are presented for age prediction. One such pipeline involves using segmentation-calculated cardiac statistics for age-prediction. Age-prediction via cardiac statistics is powered via a logistic classification model. Such a logistic classification approach enables transparent reporting of model interpretation via black-box methods such as calculation of SHAPly scores. Furthermore, we present a fully deep learning-based pipeline for age classification. Through use of raw video frames, we determine that convolutional neural networks can predict fly age with high accuracy and specificity. Additional detail for both methods is provided in the Method section.

### Representative output from our deep learning pipeline

**Figure 2:**
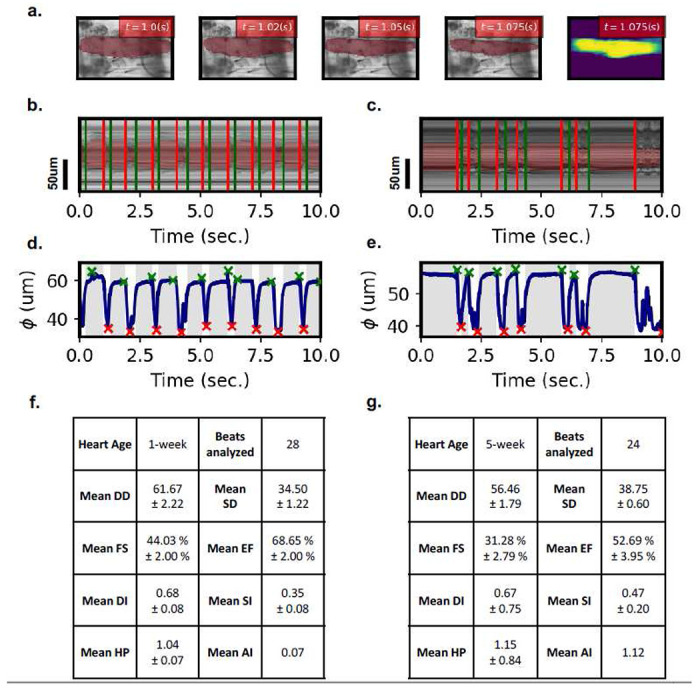
Representative output of neural network. **(a)** Select frames extracted from cardiac video, with overlaid mask from neural network denoting heart walls. **(b, c)** Using annotated frames, an annotated mechanical mode (M-Mode) image is generated. **(d, e)** Detected heart masks are used to calculate average stationary diameter on a per frame resolution. **(b, d, f)** represent generated M-Mode, time-series beating pattern, and calculated cardiac parameters for a representative 1w male heart. **(c, e, g)** represent generated M-Mode, time-series beating pattern, and calculated cardiac parameters for a representative 5w male heart. Green (start of DI) and red (end of DI) lines are annotated in **(b, c)** and as gray periods in **(d, e)**. Times of max contraction (red) and max relaxation (green) is annotated by x indicators in **(d, e)**.

**Figure 3:**
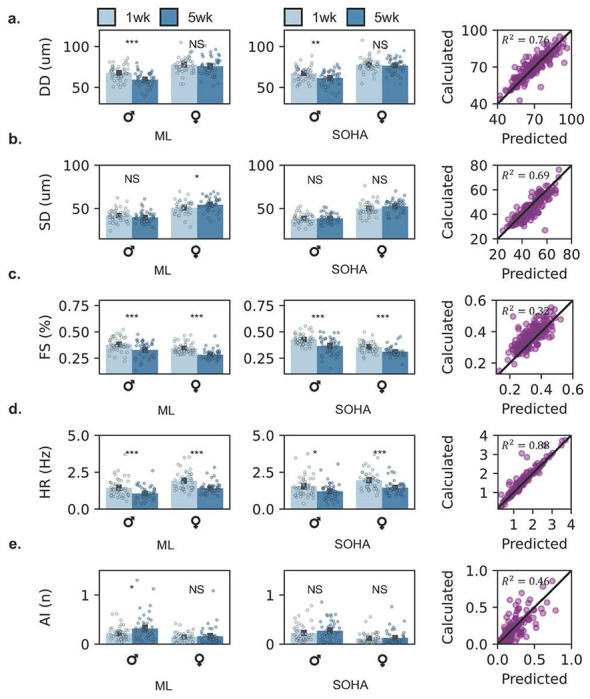
Deep learning recovers aging phenotypes in Drosophila cardiac model. **(a)** Diastolic diameter, in micron, calculated by our model (left), SOHA (right), and agreement between two datasets (right). **(b)** Systolic diameter, in micron, calculated by our model (left), SOHA (right), and agreement between two datasets (right). **(c)** Radial contractility (fractional shortening), in percentage, calculated by our model (left), SOHA (right), and agreement between two datasets (right). **(d)** Heart rate, in Hertz, calculated by our model (left), SOHA (right), and agreement between two datasets (right). **(e)** Beating dysrhythmia (arrythmia index) calculated by our model (left), SOHA (right), and agreement between two datasets (right). All error bars report ± SEM. Age-dependent statistics compared with paired t-Test statistics. Statistics are calculated via the use of a restricted ROI, selected by a trained user.

**Figure 4:**
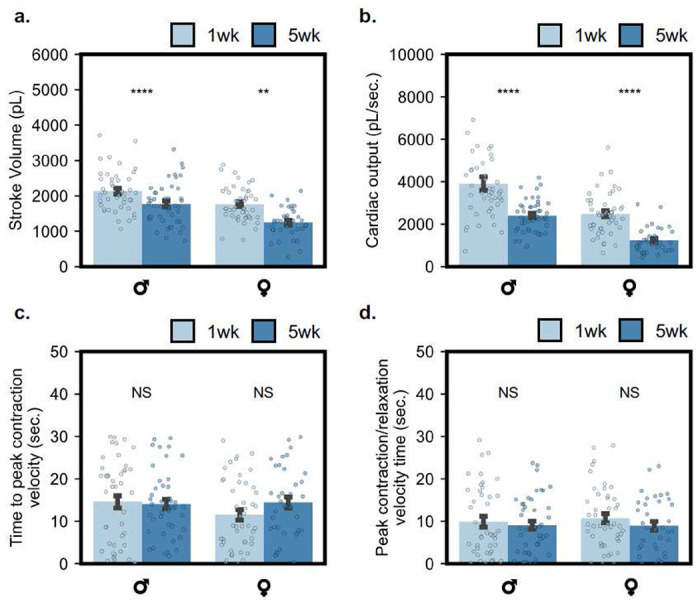
Contractile dynamics detected by deep learning. **(a)** Stroke volume for male and female aging groups is depicted, in picolitres. A significant reduction in stroke volume is exhibited with aging in both genders. **(b)** Cardiac output visualized via integration of per-beat stroke volumes and normalized by beat-times, measured in pL s-1. Aging depicts a strong reduction in cardiac output. **(c)** Time to peak contraction (negative) heart-wall velocity visualized for aging. **(d)** Time between peak contraction (negative) and relaxation (positive) velocities visualized for aging. Both **(c)** and **(d)** reflect no significant change with aging. All error bars report ± SEM. Age-dependent statistics compared with paired t-Test statistics.

Fig. 2 demonstrates a representative output from our deep learning pipeline. This includes frame-by-frame heart wall output, visualized beating patterns, time-resolved morphological data, and calculated cardiac statistics on a representative level. Heart morphology is captured on a per-frame basis, denoted by overlaid red layers (Fig. 2a), as indicated in the methods section. Qualitative inspection suggests excellent agreement between detected heart regions and ground truth through all stages of the cardiac cycle. Fig. 2b, d, f present representative model outputs for a 1-week male heart. Alternatively, Fig. 2c, e, g present representative model outputs for a 5-week male heart. We trace the annotation over time at a single vertical slice, creating a mechanical mode (M-Mode) image, demonstrated in Fig. 2b, c. Again, one may note excellent agreement between annotated and ground truth MMode, indicating excellent capture of the heart wall dynamics. Furthermore, periods of diastolic and systolic intervals are automatically labeled with red and green vertical lines. Fig. 2d, e depicts time-resolved beating patterns constructed via measurement of heart diameter through segmented heart walls. Average diameter is calculated by measuring each vertical pixel-slice distance in our frame-by-frame heart walls. This is further discussed in the Methods section. The time-resolved beating patterns clearly demonstrate differences in contractility and arrhythmia, making neural-network captured aging phenotypes clear on a representative basis. These are further quantified in tabulated cardiac statistics for a single heart (*n* = 1), made available in Fig. 2f, g. These aging phenotypes are presented for a larger-scale study in Figs. 3, 4.

### Experimental validation of Drosophila aging model

A study investigating cardiac aging phenotypes was performed on a cohort population. We demonstrate that our model can capture aging phenotypes presented in existing literature with a fraction of the effort required from canonical analysis techniques (SOHA). We calculate a full suite of cardiac statistics including diastolic diameter (Fig. 3a), systolic diameter (Fig. 3b), fractional shortening (Fig. 3c), heart rate (Fig. 3d), and arrythmia index (Fig. 3e). Furthermore, we provide select temporal statistics including heart period, diastolic intervals, and systolic intervals in the SI (Supplementary Fig. 2). Fig. 4 presents model-detected contractile dynamics, including aging phenotypes in spatial and temporal modes. Supplementary Fig. 3 depicts heart fractional shortening and heart period, as a function of gender and age at the beat-level.

### Deep learning recovers aging phenotypes including contractile dynamics in a Drosophila cardiac model

We find that expected directional trends in cardiac function and arrythmia are captured successfully across aging groups and genders (Fig. 3). Spatial (cardiac morphology) associated statistics are first discussed. We demonstrated a statistically significant decrease in diastolic diameter in male aging groups and no change in diastolic diameter across female aging groups, with agreement between experimental machine-learning (ML) and SOHA groups. Systolic diameter demonstrates little variance across aging groups with agreement between ML SOHA groups, except for a weak statistically significant difference in the ML female aging group (*p* = 0.049). Furthermore, we observe a strong statistically significant decrease of cardiac function (fractional shortening) across both male and female aging (*p* < 0.001). *R*^2^ measurements between ML and SOHA data are high for diastolic and systolic diameter measurements, at *R*^2^ = 0.76 and *R*^2^ = 0.69, respectively. However, *R*^2^ is low for fractional shortening measurements at *R*^2^ = 0.32. We demonstrate a strong reduction in cardiac function with age across both male and female groups in ML and SOHA studies, as expected. This agrees with morphological parameters quantified in Fig. 4, with a statistically significant reduction in stroke volume (Fig. 4a) and cardiac output (Fig. 4b). Methods for calculating all discussed parameters are made available in the Methods section.

Next, we discuss temporal associated cardiac statistics. We find that our model can accurately capture changes in heart rate, with expected significant differences across aging groups in both ML and SOHA datapoints. Similar agreement is presented in studied heart period, diastolic and systolic interval data across ML and SOHA groups. The model captures an accurate evolution of cardiac arrythmia in female aging data in both ML and SOHA groups but exhibits an unexpected significant difference in male aging groups. *R*^2^ measurements between ML and SOHA data are high for temporal statistics including heart rate, heart period, diastolic interval, and systolic intervals, at *R*^2^ = 0.88, *R*^2^ = 0.91, *R*^2^ = 0.89, and *R*^2^ = 0.63, respectively. However, *R*^2^ is low for arrythmia index measurements at *R*^2^ = 0.46. We observe a small increase in cardiac arrythmia with aging in both ML and SOHA groups. While heart period and derivative temporal parameters demonstrate statistically significant shifts with aging, changes in rhythmicity and contractile latency are not exhibited with aging (Figs. 3, 4). Latency is quantified in Fig. 4c, d, concluding that no significant difference in latency to peak contraction and latency between peak contraction to peak relaxation is expressed in our aging model across aging in gender-separated data. This signals agreement with previously presented results in measured arrythmia shifts with aging.

We also demonstrate our ability to capture beat-level contractile and temporal dynamics in beating patterns. In both male and female groups, we see a large flattening across 1-week and 5-week fractional shortening distributions, indicating a large increase in contractility variance (Supplementary Fig. 3 a, b). Furthermore, a general decrease in contractility with aging is depicted on a beat-level, as expected from cohort-level data. The reverse trend is true in heart period distribution, with flattening across both male and female aging groups but an increase, on average, in beat length (Supplementary Fig. 3c, d). Furthermore, we employ beat-level dynamics to detect brachy- and tachycardia arrythmias. Supplementary Fig. 4 quantifies prevalence and significant shifts in exhibited tachy- and brachy- cardiac arrythmia across our aging model. We find a significant decrease in average SI length during tachycardiac events for female specimen with aging (Supplementary Fig. 4). Additionally, we observe a significant increase in average DI length during brachycardiac events for both male and female specimen through aging. We note an exceptionally small *n*-value at *n* = 3 for brachycardiac events in young female hearts. We visualize cohort-level and beat-level data for SI and DI distributions (Supplementary Fig. 4c). Beat-level data suggests a widening distribution in both SI and DI for aging male and female specimen.

### Prediction of aging in Drosophila data

**Figure 5:**
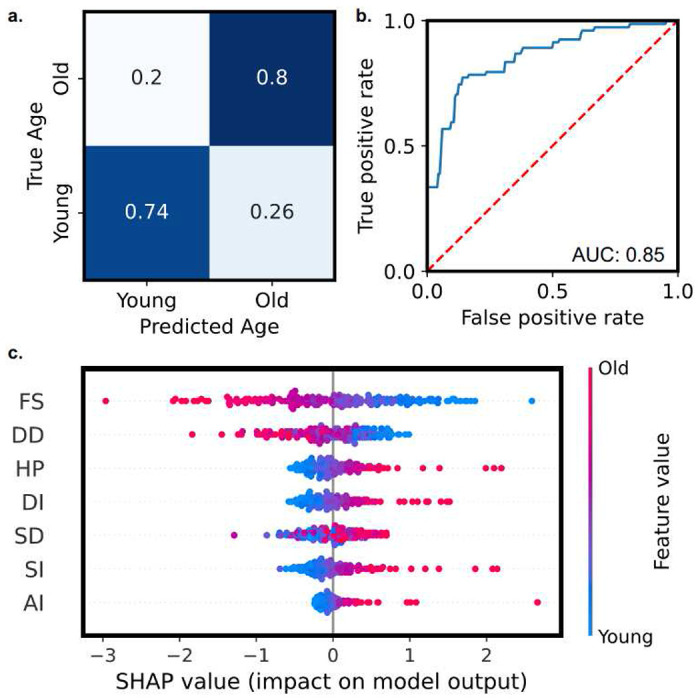
Machine learning classification of aging data. Logistic classification models enable accurate prediction of fly age from model-calculated cardiac statistics. **(a)** Heatmap of test-dataset predictions, indicating high agreement. **(b)** Average ROC-curve of all folds from presented model, reporting a mean AUROC of 0.85. **(c)** Physiological feature relevancies for predicting fly age, as determined by SHAP study. SHAP study suggests large dependence on fractional shortening (FS) in aging.

**Figure 6:**
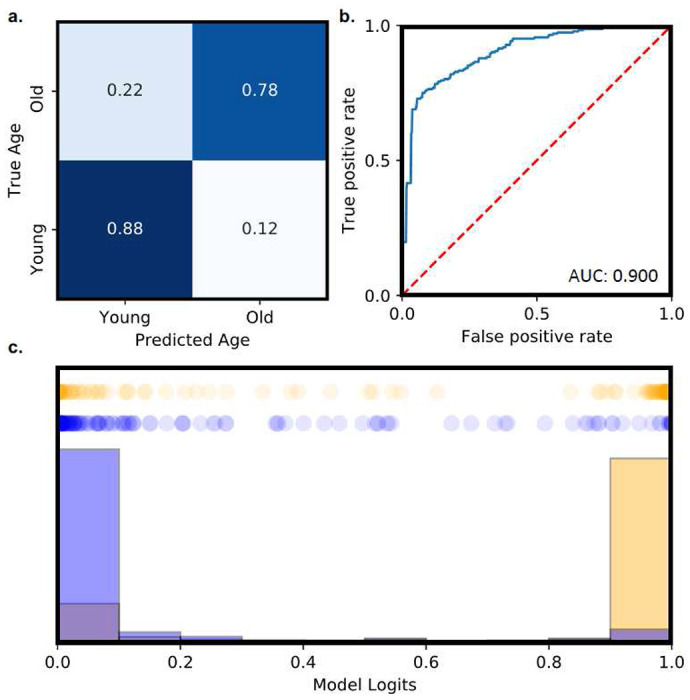
Deep learning classification of aging videos (n = 497). Deep learning models enable accurate prediction of fly age from only video data. **(a)** Heatmap of test-dataset predictions, indicating high agreement. **(b)** Average ROCcurve of all folds from presented model, reporting a mean test accuracy of 83.3% and mean AUROC of 0.900. **(c)** Model class log-likelihood distribution for investigated test predictions.

We investigate the use of machine learning approaches for age classification in *Drosophila* datasets (Figs. 5, 6). We apply logistic regression for age classification (Fig. 5). We note high accuracy in experimental classification, with an accuracy of 77.1% and AUROC of 0.85 (Fig. 5a, b). We observe the highest inaccuracies to be found in old hearts falsely classified as young. The presented results suggest that cardiac statistics are accurate predictors of aging phenotypes. We demonstrate the use of SHAP values for assessing drivers of aging function, enabling interpretable machine learning (Fig. 5c). Descriptors are sorted in predictive importance, from top to bottom. Thus, we note that SHAP-methods determine fractional shortening (FS) as a main predictor of fly age, followed by diastolic diameter (DD).

Using a deep convolutional neural network, we train a video classifier for assessing fly age from cardiac video without knowledge of cardiac parameters. Results of this technique are presented in Fig. 6. The constructed model architecture along with data preparation are discussed in the Methods section. Using a k-fold cross-validation procedure, we perform experimental validation on *n* = 497 cardiac samples, with group breakdowns of *n* = 118 for 1wm, *n* = 156 for 1wf, *n* = 121 for 5wm, and *n* = 98 for 5wf. We observe an average AUROC score of 0.90 (Fig. 6b) and an average accuracy of 83.3%, demonstrating excellent performance, further depicted in the confusion matrix (Fig. 6a). Qualitatively, we note excellent separation of age likelihood as determined by our model, with a high separation between young and old likelihoods (Fig. 6c). The presented results suggest a neural architecture can recover fly age from cardiac video based on spatial data from select frames. We note that the highest number of inaccuracies found in young hearts falsely predicted as old (22% of samples).

Overall, we determine that deep learning classification models can model aging directly on raw cardiac video with a high accuracy, suggesting such models may possess capabilities for capturing morphological or rhythmic features in image pairs. Furthermore, we determine that both cardiac parameters and videos can be applied for highly accurate aging prediction in *Drosophila* models.

## Discussion

We present methods for the machine and deep-learning analysis of Drosophila optical cardiac videos. We find that deep segmentation models can accurately recover important contractile and temporal dynamics in *Drosophila* heart beating patterns. Our approach allows us to bypass the time-consuming human involvement required in existing canonical software such as SOHA^13^. Moreover, our automated machine-learning analysis will be helpful to erase human errors in marking heart edges under contraction and relaxation conditions. This is one of the vital steps for automated analysis as three of the main cardiac parameters, including diastolic and systolic diameters as well as fractional shortening (cardiac performance) depends on accurately marking cardiac edges. Such cardiac edge marking is automated in Dong *et al.*^14^ for OCM and Klassen *et al.*^16^ for fluorescent microscopy, but yet to be automated in an optical microscopy approach. Dong *et al.*^14^ provides utilities for heart beat calculation. We provide a full suite of cardiac statistics that may be automatically calculated using our deep model. The presented code can readily provide calculated cardiac statistics including diastolic and systolic diameters/intervals, fractional shortening, ejection fraction, heart period/rate as well as quantify heartbeat arrhythmicity. Our model may be applied on readily available consumer hardware. In our study, we employ the Tesla P100, but suggest the model can be used with lower-end, consumer graphics models. Our method may potentially aid researchers with a higher fidelity, reproducible, and more automatic approach to *Drosophila* cardiac modeling beyond the capabilities of human technicians.

We use a 2D deep segmentation model to generate heart-wall segmentations on a frame-by-frame basis. We find that the employed segmentation model allows us to recover accurate heart-wall segmentations, thus, recover accurate beating patterns and cardiac statistics on a frame-by-frame resolution. Using our deep learning method, we note fly aging expresses significant reduction in cardiac function (contractility) and an increase in cardiac dysrhythmia. Similarly, our model detects significant changes in aged heart rate and heart period, as well as underlying parameters including diastolic and systolic intervals. Furthermore, such annotations open opportunities in precise time-resolved study of *Drosophila* cardiac morphologies in optical micrography assays (Fig. 2). Such analysis is not possible using canonical analysis software. For example, deep learning assisted modeling of congenital muscular dystrophies in *Drosophila* cardiac morphologies may reveal unique physiological information^30,31^.

To our knowledge, this is the first platform for deep learning assisted segmentation applied to standard high-resolution, high-speed optical microscopy of *Drosophila* hearts and is the first to quantify all relevant parameters, including directly quantifying ejection fraction. Cited works such as Ouyang *et. al*^26^ report a limited amount of cardiac parameters and provide ejection fraction as a model-derived value on human echocardiography data. Ouyang *et. al* studied failing hearts as a contrasting model to demonstrate differences in beat-level ejection fraction but did not include age dependence. Similarly, Lee *et. al*^15^ report segmentation on *Drosophila* OCM video, with fewer provided cardiac parameters including heart-rate, ESD, EDD, and FS, with some discussion on aging phenotypes. We further enable understanding of contractile dynamics via our beat-level capabilities (Figs. 3, 4, Supplementary Fig. 3). Through per-frame analysis, we quantify contractility through measurement of morphological parameters quantifying stroke performance (Figs. 3, 4) and latency (Fig. 4). We note significant reduction in spatial beating modes (FS, SV, CO) with aging, but little to no dependence on aging for temporal beating character (AI, latency) (Fig. 4). However, we do note significant shifts with aging in beat lengths, indicated in modeling of HP and derivative parameters. Beat-level investigation elucidates per-beat information regarding cardiac arrythmia’s including tachycardia and brachycardiac arrythmias (Supplementary Fig. 3). We observe a significant, large increase in DI length during brachycardiac events in 5wk male flies.

We demonstrate capabilities of predicting fly age using experimentally calculated cardiac statistics with excellent agreement (Fig. 5). We also use a 2D video classification model to predict fly ages between 1-week and 5-week groups (Fig. 6). The ability to classify age via both raw video frames (Fig. 6) and cardiac statistics suggests that experimentally calculated cardiac statistics are physiologically salient and model age dependence. Additionally, the ability to predict age with only video frames suggests that deep learning models can capture morphological and rhythmic patterns in *Drosophila* cardiac video data. This has important implications for detecting phenotypes mimicking or delaying aging of *Drosophila* hearts. To our knowledge, we are the first work to determine that deep neural networks can capture such cardiac physiological features of the heart that induce aging in cardiac videos. In the future, such classification could be extended to classify healthy and compromised hearts in mutant studies and may include further quantification through methods such as GradCAM^32^.

Current limitations include validation of parameters including calculation region of interest and suitable confidence thresholds. In the future, we hope to overcome this limitation. We report preliminary results in the Supplementary Fig. 3 and Supplementary Fig. 4 of using our trained segmentation approach without specifying a region of interest for morphological calculation (referred to as “No ROI”). Furthermore, we homogenously employ a pre-selected threshold, enabling a user to analyze samples with no human input or supervision. Presented results demonstrate excellent agreement in temporal statistics, including high accuracy in heart rate/period, diastolic and systolic intervals (with comparison to data collected via SOHA software), but strong disagreement in the arrythmia index. Spatial statistics, however, demonstrate strong disagreement in systolic diameter, driving further disagreement in fractional shortening. We propose further developments in dataset size, domain adaptability, and self-supervision may lead to stronger spatial performance in the case of completely hands-free usage.

Future applications of the discussed techniques include enabling deep learning assisted studies of cardiac mutation models, other small animal models (e.g., commonly studied zebrafish and mice models), and parameter calculation in human heart models. Furthermore, quantification of measured uncertainty techniques may be applied to qualify certainty of heart analyses.

Massive volumes of *Drosophila* cardiac data collected in the lab necessitate advanced methods for automated analysis of cardiac physiologies and morphologies on a beat-by-beat basis. In summary, we evaluate the use of deep segmentation models for high-fidelity analysis of cardiac physiologies in high-speed *Drosophila* cardiac optical recordings. We demonstrate that the presented deep segmentation models can be applied for accurately expressing known *Drosophila* phenotypes in aging across male and female 1-week and 5-week groups. Furthermore, we demonstrate that developed deep video classification methods can be successfully applied to Drosophila fly age-classification using only video clips with exceptional accuracy. We hope these models can be applied in the future to expedite laboratory analysis and power next-generation *Drosophila* cardiological model analysis.

## Methods

### High-speed cardiac recording

Physiological cardiac parameters such as heart rate, heart period, diastolic and systolic diameters, diastolic and systolic intervals, cardiac rhythmicity, and cardiac performance (% fractional shortening) will be determined for each fly group to detect cardiac defects using established protocols^4–7^. To avoid any circadian variability in cardiac function, all assays will be performed between ZT4 and ZT8. Briefly, in these semi-intact heart preparations, nerve input is eliminated so that endogenous pacing can be observed. Direct immersion optics is used in conjunction with a digital high-speed camera (up to 150 frame/s, Hamamatsu EM-CCD) to record contraction movements using the image capture software HC Image (Hamamatsu Corp.).

### Dataset curation

A standard library of high-speed cardiac optical recordings is procured for training. We employ a total of 54 training videos, with an 85%/15% train/validation split. Training videos are captured in grayscale format with a total of 500 frames per video from a complete 6000 frames, indicating 2.5 seconds of video. Training videos include 40 videos with wildtype and 14 videos with genetically engineered flies. Researchers used the Computer Vision Annotation Tool (CVAT) software to produce high-quality masks on a per-frame basis. Annotations spanned heart walls that were clearly identifiable – annotations were clipped in the presence of pericardial occlusion, change of heart regions, and unclear tissue. Annotation masks were reviewed for accuracy before usage in deep-learning experiments. Testing videos are captured in identical formats. We procure 177 videos for experimental validation, with *n* = 46 for 1-week males, *n* = 43 for 1-week females, *n* = 44 for 5-week males, and *n* = 44 for 5-week females.

### Deep-learning development and training

Model design was performed in Python using the PyTorch deep learning framework^29^. Our study employs a modified Attention-UNet architecture for semantic segmentation^22^. The model contains a symmetric encoder/decoder architecture containing 8, 16, 32, 64, and 128 filters, employing a rectangular convolutional kernel. Validation loss was optimized via Dice Loss^33^. Our model employs a random weight initialization and the Adam^34^ optimizer and a 16-image batch size. For experimental testing, the model epoch with the lowest validation loss was employed. During the training process, the neural network samples data via a data point’s corresponding diameter, as measured via its human-annotated mask. 75 frames per measured diameter are randomly sampled to be included in the training dataset – this is done to minimize class imbalance in our segmentation task. After frame sampling, 3D samples are constructed by appending frames at (*t* − 4, *t*, *t* + 4) indices, allowing us to encode temporal information in 3D convolutions. After sampling and augmentation, the model used a total of 114750 images for training with 19350 images used for validation. The model was trained for a total of 30 epochs, yielding a total of approximately 30 compute hours. Models were trained on a Tesla P100 GPU on the UAB Cheaha supercomputer.

### Calculation of cardiac parameters

Once a video has been selected for analysis, each frame *t* is converted into a three-channel image with the *t* − 4 frame and the *t* + 4 frame. Images are then processed via neural network. On a Tesla P100, we estimate this process to take approximately 103 seconds for a video with 5990 frames (58.16 FPS). From sigmoidal activations, the user determines an acceptable threshold and region-of-interest (ROI) through visual confirmation. Using provided values, average heart diameter for each frame is measured. Processing codes for the identification of diastolic intervals (DI) and systolic intervals (SI) is provided in the paper repository (GitHub). The diastolic diameter (DD) for each DI is taken to be the largest diameter attained during the DI. Similarly, the systolic diameter (SD) for each SI is taken to be the smallest diameter attained over its duration. We derive additional cardiac parameters from the collected DD, SD, DI, and SI statistics. Equations for fractional shortening (FS), ejection fraction (EF), heart period (HP), heart rate (HR), and arrhythmia index (AI) can be found in literature^6^. Stroke volume and cardiac output calculations follow those in Klassen *et al.*^16^, along with peak contraction velocity and peak contraction to relaxation latencies^16^. Velocity information is procured via numeric differentiation of frame-level time-resolved diameter data. Extraction of beat-level data enables capturing of SI and DI arrythmias, referred to as tachycardiac and brachycardiac arrythmias. After identifying all SI and DI at the beat-level, tachycardiac data is selectively filtered from all SI events with a length over 0.5 sec. Brachycardiac events are extracted from DI data with a length over 1.0 sec. These filtering parameters are extracted analysis performed by Occur *et al.*^4^.

### Experimental validation

We procure high-speed videos of *n* = 46 for 1-week males, *n* = 43 for 1-week females, *n* = 44 for 5-week males, and *n* = 44 for 5-week females to experimentally validate our model. For each heart, we calculate time-resolved beating patterns and cardiac statistics using our deep-learning approach. For each heart, an end-to-end analysis takes approximately 2 minutes. Next, each heart was identically examined in the canonical software for small-animal cardiac analysis, titled SOHA^13^. We employ the SOHA software for experimental validation of our model using blind data. Statistics comparing age-dependent phenotypes are calculated using generally available Python packages. For comparison of aging groups, we calculate t-Test significance values. Furthermore, for a quantitative view of our model performance, we calculate pairwise coefficient of determination information (*R*^2^) score via the Scikit-Learn Python library^35^. A close agreement between deep-learning calculated data and SOHA data indicates high model performance.

### Classification of *Drosophila* age

Calculated cardiac statistics are exported from experimental studies. A dataset is labelled with cardiac statistics and corresponding fly age (1-week, 5-week). We produce a predictive model combining calculated cardiac statistics and fly age via logistic classification. To fit this logistic model, we employed the Scikit-Learn Python library. Our logistic model was fit on experimentally predicted DD, SD, FS, DI, SI, HP, and AI parameters. A *k*-fold cross-validation with *k* = 5 folds was used to evaluate model performance. Using such a configuration achieved a high testing accuracy with our model, as quantified in the Results section. To elucidate the connection between cardiac physiological parameters and age-dependence, we employ the SHapley Additive explanations (SHAP)^36^ technique. Doing so enables model transparency and insights into main determinants of aging phenotypes.

We also investigate the use of deep learning models for the classification of *Drosophila* age from cardiac recordings. For this task, the sum of the squared difference of each pixel between a frame *t*_0_ and frame t is saved as timeseries data and are normalized between 0 and 1. This data is binarized with a threshold of 0.5. The resulting timeseries is composed of alternating regions of consecutive ones and consecutive zeros. The frame corresponding to the center of each region was saved until 96 frames had been collected. This along with the duration between each frame was taken as the input for the model. The model passes the 96-frame clip through 3 convolution blocks each consisting of two 2D convolutional layers followed by a max pooling layer. The result is then flattened and combined with the duration data before being passed into three dense layers and a final sigmoid output layer. Our dataset consisted of *n* = 118 for 1-week males, *n* = 156 for 1-week females, *n* = 121 for 5-week males, and *n* = 98 for 5-week females. Again, we utilized k-fold cross-validation to evaluate model performance with *k* = 5 folds.

## Data Availability

Data used within this paper and connected findings of this study are available from the corresponding author upon request.

## Significance

- Current analysis of *Drosophila* cardiac recordings is capable of limited cardiac physiological parameters and are error-prone and time-consuming.
- We present the first deep-learning pipeline for high-fidelity automatic modeling of *Drosophila* contractile dynamics.
- We present methods for automatically calculating all relevant parameters for diagnosing cardiac performance in aging model.
- Using the machine and deep learning age-classification approach, we can predict aging hearts with an accuracy of 83.3% (AUC 0.90) and 77.1% (AUC 0.85), respectively.
